# Assessing the Feasibility and Preliminary Effects of a Web-Based Self-Management Program for Chronic Noncancer Pain: Mixed Methods Study

**DOI:** 10.2196/50747

**Published:** 2024-05-03

**Authors:** Pascale Marier-Deschenes, Anne Marie Pinard, Laura Jalbert, Annie LeBlanc

**Affiliations:** 1 Laval University Medicine Faculty Québec, QC Canada; 2 CIRRIS, Centre interdisciplinaire de recherche en réadaptation et intégration sociale Québec, QC Canada; 3 CHU de Québec Université Laval Québec, QC Canada; 4 VITAM Centre de recherche en santé durable Québec, QC Canada

**Keywords:** persistent pain, eHealth, self-paced intervention, web-based program, evidence based, web based, self-management, pain, chronic pain, mixed methods study, pain treatment, pain education

## Abstract

**Background:**

In Canada, adults with chronic noncancer pain face a persistent insufficiency of publicly funded resources, with the gold standard multidisciplinary pain treatment facilities unable to meet the high clinical demand. Web-based self-management programs cost-effectively increase access to pain management and can improve several aspects of physical and emotional functioning. Aiming to meet the demand for accessible, fully automated resources for individuals with chronic noncancer pain, we developed a French web- and evidence-based self-management program, *Agir pour moi* (APM). This program includes pain education and strategies to reduce stress, practice mindfulness, apply pacing, engage in physical activity, identify and manage thinking traps, sleep better, adapt diet, and sustain behavior change.

**Objective:**

This study aims to assess the APM self-management program’s feasibility, acceptability, and preliminary effects in adults awaiting specialized services from a center of expertise in chronic pain management.

**Methods:**

We conducted a mixed methods study with an explanatory sequential design, including a web-based 1-arm trial and qualitative semistructured interviews. We present the results from both phases through integrative tables called joint displays.

**Results:**

Response rates were 70% (44/63) at postintervention and 56% (35/63) at 3-month follow-up among the 63 consenting participants who provided self-assessed information at baseline. In total, 46% (29/63) of the participants completed the program. We interviewed 24% (15/63) of the participants. The interview’s first theme revolved around the overall acceptance, user-friendliness, and engaging nature of the program. The second theme emphasized the differentiation between microlevel and macrolevel engagements. The third theme delved into the diverse effects observed, potentially influenced by the macrolevel engagements. Participants highlighted the features that impacted their self-efficacy and the adoption of self-management strategies. We observed indications of improvement in self-efficacy, pain intensity, pain interference, depression, and catastrophizing. Interviewees described these and various other effects as potentially influenced by macrolevel engagement through behavioral change.

**Conclusions:**

These ﬁndings provided preliminary evidence that the APM self-management program and research methods are feasible. However, some participants expressed the need for at least phone reminders and minimal support from a professional available to answer questions over the first few weeks of the program to engage. Recruitment strategies of a future randomized controlled trial should focus on attracting a broader representation of individuals with chronic pain in terms of gender and ethnicity.

**Trial Registration:**

ClinicalTrials.gov NCT05319652; https://clinicaltrials.gov/study/NCT05319652

## Introduction

### Background

The prevalence of chronic pain, including chronic cancer pain in adults, is estimated to be between 18% and 21%, with severe repercussions for all aspects of the lives of those affected, their families, and society [1-3]. Chronic pain affects patient-perceived health status and psychological functioning; decreases energy levels; and hinders engagement with physical, emotional, cognitive, and social activities [3-6]. These impacts can strain familial and social relationships and affect work performance [7]. Living with chronic pain often involves increased medical expenditures and detrimentally affects one’s financial well-being [3,6]. In addition, the wait for services is not without added consequences to these repercussions, with long wait time (12-30 months) being associated with further deterioration in pain-related interference, psychological distress, and pain acceptance [3,8-10].

In Canada, adults with chronic noncancer pain face a persistent insufficiency of publicly funded resources, with the gold standard multidisciplinary pain treatment facilities being unable to meet the high clinical demand [1,2,11]. Since 2019, the Canadian Task Force has reaffirmed the necessity to implement equitable and innovative ways to deliver health interventions in a timely manner in the public network [12,13]. Web-based self-management programs that include exercise, sleep hygiene, pacing, and a healthy lifestyle are endorsed as part of the therapeutic considerations and recommendations for chronic noncancer pain management [14]. These programs have shown an impact on patients’ pain intensity, pain interference [15,16], anxiety [15,17], depression [17,18], stress [18], catastrophizing, and self-efficacy [19].

The lack of accessible and reliable unguided web-based self-management programs tailored to French-speaking individuals with chronic noncancer pain is a significant yet solvable health services gap. Over the years, individuals with lived experience, organizations, and researchers have stressed the relevance and importance of actively involving patient partners in the health intervention development process [20-25]. Therefore, a novel French web-based self-management program for chronic noncancer pain developed in collaboration with individuals with lived experience could meet the specific needs of French-speaking individuals [26].

### Objectives

This study aims to (1) assess the feasibility and acceptability of the *Agir pour moi* (APM) self-management program and trial procedures and (2) explore preliminary outcomes in individuals living with chronic noncancer pain.

## Methods

### Study Design

We conducted a mixed methods sequential explanatory study consisting of a single-arm, pre- and postintervention trial, followed by qualitative, semistructured interviews with adults experiencing chronic noncancer pain and awaiting services from a center of expertise in chronic pain management [27-29]. We registered the trial at ClinicalTrials.gov (NCT05319652) and followed the CONSORT-EHEALTH (Consolidated Standards of Reporting Trials of Electronic and Mobile Health Applications and Online Telehealth) and guidelines for reporting nonrandomised pilot and feasibility studies [30,31].

### Ethical Considerations

The University Hospital Centre (CHU) de Québec-Université Laval Research Ethics Board approved the study (#2023-6312).

### Knowledge Users’ Involvement

A total of 7 individuals with lived experience of chronic pain, 5 health care professionals with experience and expertise in chronic pain management, 3 medical students, and 1 graphic designer contributed to the program’s codevelopment. We engaged in web-based, phone, or email conversations over a period of 1.5 years. All knowledge users could contribute to various aspects of program development, including its identity (eg, colors, logo, and name), structure (eg, lesson sequence, content organization, and navigation), content (eg, self-management strategies, theoretical content, and testimonials), and learning modalities and behavior change techniques (eg, personal plans, reflective activities, and interactive scenarios). These knowledge users were not further involved across the duration of the trial, but individuals with lived experience initially guided the team toward reducing the questionnaire burden to a minimum for the participants.

### Study Setting, Participants, and Recruitment

We recruited participants from the center of expertise in chronic pain management waitlist at the CHU de Québec-Université Laval. This center provides superspecialized services intended for complex chronic pain cases requiring a technical platform and multidisciplinary team. Most individuals referred to such centers experience significant impairments, including high pain levels interfering with their daily life, moderate to extremely severe depression, and pain-related sleep disturbance. Most of them take prescription analgesic medication and have already consulted different types of health care professionals [2].

Using the center’s assigned priority level, between June and August 2022, we sent 500 invitation letters to adults (aged >18 years) with chronic noncancer pain (for >3 months) unlikely to receive services within the next 6 months. Interested individuals were to email us to set an eligibility interview, confirming that they understood French, had access to a computer and high-speed internet, had not started a new treatment for pain within the last 1 month and agreed to notify us before starting a new one, were available for the duration of the study, and were able to provide informed consent. We excluded individuals who participated in a chronic pain self-management program within the last year or those who were scheduled for surgical treatment within 6 months. Following the assessment for eligibility, a research team member explained the study procedures and recorded verbal informed consent.

### Intervention

The codevelopment of the APM self-management program (thereafter APM or the program) is detailed [32] and available in the study by Marier-Deschenes et al [33]. Briefly, we designed a cognitive behavioral therapy (CBT)–centered, web-based self-management program that would enable participants to develop their self-management skills autonomously (eg, goal setting), practice suggested self-management strategies (eg, pacing), and sustain new behaviors (eg, respect of limits). Despite the diverse nature of our targeted population, the proposed self-management strategies to explore and develop are mostly universal, spanning areas such as managing thoughts and emotions, gradually resuming physical activity, practicing pacing, and adopting good sleep hygiene. The program is self-guided (ie, unguided) in that it provides the same information as face-to-face programs offered in tertiary pain clinics but without therapeutic support from health care professionals [34]. It is structured around weekly lessons over an 8-week period with content that encompasses 26 different behavior change techniques [35] and a downloadable personal plan (Table 1).

**Table 1:** Summary of Agir pour moi (APM) program’s topics and self-management strategies with associated content.

Week	Topics and strategies	Lesson headers
Foreword	What does APM offer?	What is self-management? Who is this program for? Will you have less pain? Key attitudes to adopt How to navigate the program?
Week 1	Introduction	What is chronic pain? Are you ready for self-management? How to set specific, measurable, appealing, realistic, and time-bound (SMART) objectives?
Week 2	Engage in well-being activities	Reduce stress Experience mindfulness
Week 3	Practice pacing	Follow-up on last week’s objective Evaluate your energy expenditures
Week 4	Practice pacing, continued	Follow-up on last week’s objective Planning your weeks
Week 5	Engage in physical activity	Follow-up on last week’s objective Stretching exercises Engage in physical activity that you enjoy
Week 6	Take care of your thoughts	Follow-up on last week’s objective Identify thinking traps Perceive the positive
Week 7	Revise your lifestyle habits	Follow-up on last week’s objective Promote sleep Adapt your diet
Week 8	Plan for the future	Reflect on previous objectives and further goals Sustain the change

The program incorporates a variety of media, including photos, infographics, interactive scenarios, tables, audio recordings, and videos. All content is fully narrated, with short audio clips accompanying written information in each lesson, ensuring accessibility in both formats. Interactive exercises such as quizzes, drag-and-drop questions, and real-life scenarios enhance understanding.

Participants were encouraged to set and track their own weekly objectives related to the topic in their personal plan, which serves as our learner’s workbook. This plan features reflective, observational, monitoring, problem-solving, and action-planning activities.

Participants were advised to allocate 60 to 90 minutes weekly for program activities. They had the flexibility to divide the lessons into multiple short sessions; completing each lesson in 1 sitting was not necessary. While participants were encouraged to follow the program sequentially, all 8-week content was readily accessible.

Following the program poses minimal health risks. The program incorporates low-intensity activities, such as stretching exercises, which might cause temporary discomfort when resumed. However, the risks associated with physical inactivity, including the development or worsening of chronic illnesses, outweigh those of gradually resuming physical activity.

### Quantitative Data Collection and Outcomes

#### Overview

Participants were assigned a log-in user ID and password for the program’s web-based platform. They completed self-reported questionnaires on the web at 3 time points: preintervention, postintervention, and 3 months after completing the program. We sent an email reminder to those who did not log in at least once a week or complete questionnaires at the appropriate time. Participants who completed all questionnaires were eligible for a random computerized drawing of 5 CAD $75 (US $55.89) gift cards. We provided participants facing technical difficulties with phone support. We used REDCap (Research Electronic Data Capture; Vanderbilt University), a secure web application, for creating and managing surveys and databases.

As this was a feasibility study, we did not perform a power calculation on measures of effect but rather aimed at estimating the number of eligible participants and the potential recruitment rate from the center of expertise in chronic pain management waitlist. Therefore, this study is not appropriately powered to assess APM’s efficacy [36].

#### Feasibility and Acceptability Outcomes

We considered the following outcomes in assessing the feasibility of the intervention and research methods and the acceptability of the program: (1) feasibility of recruitment (number of referred adults who responded to the invitation and consented to participate in the study and number of interested adults excluded based on inclusion and exclusion criteria), (2) feasibility of data collection (rate of response to and completion of the questionnaires at each time point), (3) acceptability for those who engaged with the program (mean score to the Acceptability eScale, which includes dimensions of usability and satisfaction) [37,38], and (4) engagement (number of lessons completed). Participants completing at least 6 (75%) out of the 8 weekly lessons were defined as program completers.

#### Effects Measures

We opted for the French versions of the following self-reported measures based on the recommendations of the Initiative on Methods, Measurement, and Pain Assessment in Clinical Trials [39] (Table 2).

**Table 2:** Self-reported measures.

Measures	Items, n	Constructs	Score range	High score meaning
Pain Self-Efficacy Questionnaire [40,41]	10	Individual’s conﬁdence to perform activities while experiencing pain	0-60	Better self-efficacy
Pain intensity subscale of the Brief Pain Inventory [42,43]	4	Worst, least, average, and current pain intensity	0-10	Worse pain intensity
Pain interference subscale of the Brief Pain Inventory	7	Impact of pain on general activity, mood, walking ability, normal work, sleep, relationships, and enjoyment of life	0-10	Worse pain interference
Anxiety subscale of the Hospital Anxiety and Depression Scale [44]	7	State of anxiety	0-21	Worst anxiety symptoms
Depression subscale of the Hospital Anxiety and Depression Scale	7	State of depression	0-21	Worst depressive symptoms
Pain Catastrophizing Scale [45]	13	Catastrophic thinking and maladaptive responses to pain	0-52	Worst catastrophizing
Patient Global Impression of Change Scale	1	Patient’s rating of overall improvement	1-7	Greater change

#### Statistical Analyses

We performed descriptive statistics using means (SD) for continuous outcomes and frequencies (%) for categorical outcomes. We compared pre-, post-, and follow-up intervention scores for effect measures using repeated-measures linear models. All statistical analyses were conducted using R software (version 4.3.0; R Foundation for Statistical Computing) in RStudio (version 2023.06.0; Posit PBC) [46,47]. Models were fit using lme4 (version 1.1-33; R Foundation for Statistical Computing) [48]. Model fit evaluations and assumption checks were done through visualizations using performance (version 0.10.4) [49]. Effects were considered significant when the 95% CI for the estimates did not include 0.

### Qualitative Data Collection and Outcomes

#### Overview

We formed a heterogeneous group of 20 potential participants using their Acceptability eScale and pre- and posteffect measure scores. We invited participants with positive and negative impressions of the intervention and those for whom we could observe the effects on functioning or not. From a practical perspective, we made the decision to not interview individuals who did not engage with the program at all, as they would not have been able to provide valuable insights into the program’s acceptability and feasibility. However, we conducted interviews with participants who did not complete the program; they were just not specifically selected based on this criterion. We conducted semistructured, audio-recorded, 40-minute phone interviews 5 to 7 months after the intervention. We achieved data saturation with 15 interviews (12/15, 80% women and 3/15, 20% men) and did not deem it necessary to conduct further interviews [50].

#### Data Analysis

We analyzed the transcriptions using inductive and deductive thematic analysis based on the motivational model for pain self-management [51]. The lead author read the interviews multiple times to obtain a detailed understanding, then coded them according to the research questions and with consideration for the model’s components. According to the model by Jensen et al [51], the willingness to embrace pain self-management behaviors is influenced by 2 primary factors. First, it is molded by beliefs concerning the perceived importance of these behaviors, encompassing considerations of cost/benefit ratio, learning history, and current contingencies. Second, self-efficacy, denoting personal beliefs about one’s abilities to accomplish a specific task, also plays a pivotal role in shaping the inclination toward behavior change. Furthermore, to ensure the validity of the analysis, a research associate coded and discussed 3 interviews. Then, the lead author further identified meaningful units and assembled them into descriptive categories. She analyzed, interpreted, and summarized categories into 3 explanatory themes that were then discussed among all coauthors [52].

### Integration

We addressed our study’s main objectives by integrating the quantitative and qualitative results, drawing on all relevant data. We opted for 2 approaches: a weaving approach through the narrative and 2 joint displays presenting categories and associated quotes explaining the quantitative data [53,54].

## Results

### Feasibility

**Figure 1:**
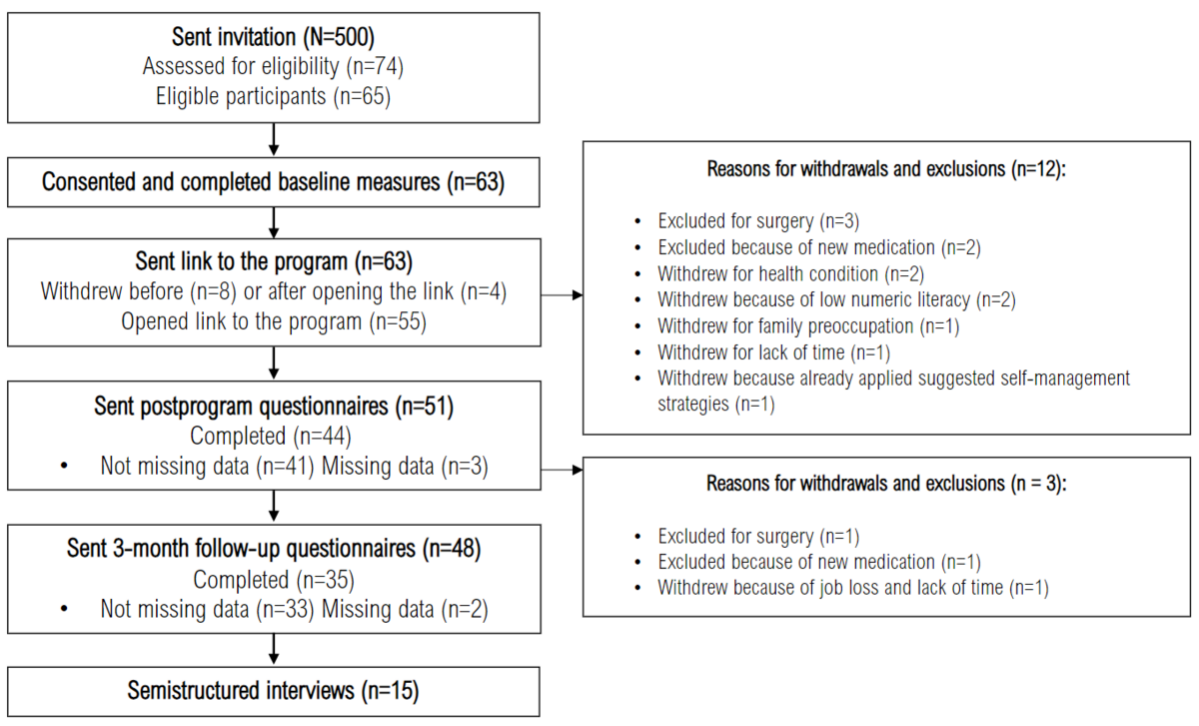
Flow diagram of the participants’ recruitment, enrollment, and engagement.

From the 500 invitations sent, 74 (15%) individuals expressed interest, of which 65 (13%) were confirmed eligible. Of these 65 eligible individuals, 63 (97%) consented to participate (Figure 1). A total of 9 (12%) of the 74 participants were ineligible due to unavailability during summer, current services from a pain clinic or rehabilitation center, lack of belief in program helpfulness, absence of computer access, low literacy level, and being unreachable. Response rates were 70% (44/63) at postintervention and 56% (35/63) at 3-month follow-up. A total of 15 (24%) of the 63 participants exited the study for reasons mostly unrelated to the intervention. Missing data were attributed to connectivity problems with the REDCap platform.

Participants were almost exclusively White (61/63, 97%) and female (44/63, 70%), with a mean age of 54 (range 24-75) years. On average, participants experienced chronic pain symptoms for 12 (SD 13.6) years, and most (34/63, 54%) had chronic musculoskeletal pain (Table 3).

**Table 3:** Participants’ sociodemographic and clinical characteristics (N=63).

Sociodemographic characteristics			Values, n (%)
**Age (years)**			
	20 to 29	2 (3)	
	30 to 39	6 (10)	
	40 to 49	13 (21)	
	50 to 59	17 (27)	
	60 to 69	19 (30)	
	70 to 79	6 (10)	
**Gender**			
	Women	44 (70)	
	Men	17 (27)	
	Prefer not to answer	2 (3)	
**Race**			
	White	61 (97)	
	People of color	2 (3)	
**Marital status**			
	Married	26 (41)	
	Living common law	20 (32)	
	Single	10 (16)	
	Widowed	3 (5)	
	Divorced or separated	4 (6)	
**Education level**			
	No certificate, diploma, or degree	3 (5)	
	High-school diploma or equivalency certificate	17 (27)	
	Apprenticeship, trades certificate, or diploma	6 (10)	
	College, CEGEP, or other nonuniversity certificate or diploma	20 (32)	
	University certificate or diploma below bachelor level	5 (8)	
	University diploma or degree at bachelor level or above	12 (19)	
**Employment**			
	Full-time	18 (29)	
	Part-time	6 (10)	
	Off work for a short or undetermined term	8 (13)	
	Off work for a long term or disabled	12 (19)	
	Unemployed	2 (3)	
	Retired	16 (25)	
	Other	1 (2)	
**Household income**			
	CAD 0-$49,999 (US $0-$37,257)	19 (30)	
	CAD $50,000-$99,999 (US $37,258-$74,515)	24 (38)	
	CAD ≥$100,000 (US ≥$74,516)	12 (19)	
	Do not know	2 (3)	
	Prefer not to answer	6 (10)	
**Duration of chronic pain (years)**			
	1 to 5	26 (41)	
	6 to 10	15 (24)	
	11 to 15	6 (10)	
	16 to 20	5 (8)	
	≥21	11 (17)	
**Type of chronic pain**			
	Chronic widespread pain (includes fibromyalgia syndrome)	24 (38)	
	Complex regional pain syndrome	8 (13)	
	Chronic musculoskeletal pain (eg, cervical pain and low back pain)	34 (54)	
	Chronic headache and orofacial pain (eg, migraine)	10 (16)	
	Chronic visceral pain	5 (8)	
	Chronic neuropathic pain	9 (14)	
	Inflammatory arthritis (eg, rheumatoid arthritis and psoriatic arthritis)	8 (13)	
	Osteoarthritis	22 (35)	
	Other	6 (10)	

^a^CEGEP : College of General and Professional Teaching.

### Acceptability

Among the 63 participants, 38 (60%) completed the Acceptability eScale. The average total score for these participants was 25.2 out of 30 (acceptability threshold=24). In Table 4, we have presented the integrated results of the quantitative and qualitative phases. The mean score for each of the 6 items from the Acceptability eScale is listed in the first column. Each item score ranges from 1 to 5, with higher scores reflecting higher acceptability. The interviews revealed a consistent theme: a program that is globally acceptable, easy to use, and engaging. Interviewees felt they could opt for what worked for them once they had experienced it completely. They would keep most of the program as is, with some potential minor improvements.

**Table 4:** Joint display of participants’ perceptions of the Agir pour moi program’s acceptability.

Quantitative results	Qualitative findings	Integrated analysis
Mean score of the ease-of-use item: 4.0/5 (SD 0.9); 5 (8%) of the 63 participants needed support at least once to log in	Ease of navigation “Everything is very easy. We can go forward, we can go back, we can resume, we can close, come back. No, everything is perfect.” [INT^a^ 7] User-friendly, except for the platform hosting the web-based program “As I was saying, when you’re a participant, and you’re logged in, I think it is super user-friendly ... It’s more when you get to the big link page, I believe that’s a little less user-friendly.” [INT 13]	The program was user-friendly once logged in, but connecting to the hosting platform could have been more intuitive.
Mean score of the strategies’ helpfulness item: 4.1/5 (SD 0.9)	Picking the tool you need “That’s it, because if you don’t have that resource, what do you do? On a day when things aren’t going well, you brood, you grumble all day long; you’re in pain, you’re angry, and you lose your patience, so if you think a little bit after having followed the program, you go back and find the tool you needed in it. Because as far as I’m concerned, the program wasn’t one singular tool; it was a toolkit, and then you take what you need.” [INT 10] “I was happy to see all this in the program, because it helped me a lot personally.” [INT 8] Adaptable to one’s situation “It’s good to have all the suggestions in there because, ultimately, we keep what suits us and works well for us. So there’s something for everyone, for everyone’s tastes, and it’s not the same stuff that works for everyone.” [INT 12] Developing further the strategies that suit you best “It’s been like a springboard to the rest of my journey, to do some reading... ... it sure helps with the perception of managing what we’re capable to manage on our own.” [INT 6]	Some strategies benefited many participants, while others only reached a minority. Interviewees latched on to at least 1 lesson that triggered something in them. They acquired something useful out of it, although not all appreciated the same strategy or strategies.
Mean score of the required time item: 4.2/5 (SD 0.7)	Objective-dependent efforts “I didn’t think it was very demanding, and after that, the rest is up to you... then you do it at your own pace, so I thought it was very appropriate.” [INT 13] Motivating and minimal effort required “Oh, for me, it wasn’t that much effort, no. Perhaps the first week’s lessons were a little longer than the others that followed. But no, it’s really not...it didn’t take me any effort, no. It was motivating.” [INT 7] A little demanding yet tangible “I think that’s what makes it interesting. Because just reading is okay, but afterward, when you do the exercise, you push yourself a bit on the spot; sometimes, you don’t really feel like doing it, but it makes it more tangible.” [INT 11]	The time and effort required to follow the program and apply self-management strategies were adequate, but interviewees highlighted the necessity to reach a certain degree of readiness to change because taking action required some investment.
Mean score of the use appreciation item: 4.3/5 (SD 0.7)	Adapting use to your schedule “As I said, we could follow it whenever we wanted. I chose the time of the day when I was in better shape. That was something I thought was very nice, you know, not having to connect at a fixed time.” [INT 15] Staying engaged “It went really well. Then, you know, you’d scroll, then you’d click, scroll, click, so that, you know, it kept you present; you had to be there... It’s not like a video you start and then lose focus. That’s very interesting too.” [INT 11] Fun program “it’s super fun.” [INT 13]	Participants appreciated navigating the program at their convenient time. It was fun to use, and the need to scroll through the content kept them engaged.
Mean score of the comprehensibility item: 4.5/5 (SD 0.6)	Very simple explanation “It’s very...it’s simple. It’s very well explained.” [INT 7]	The program presented well-explained, easy to understand information.
Mean score of the satisfaction item: 4.1/5 (SD 0.7)	Participants would recommend the program “I really enjoyed the program. I liked that there were videos, it made it more dynamic, I thought they were well done, well constructed ... I’d definitely recommend it.” [INT 12] Down-to-earth expectations, no promises “I thought the program was interesting... Knowing what’s in it, I’d do it again today... In the beginning, you don’t make any promises. In the program, it says ‘learning to live with chronic pain’, but there’s no promise; it doesn’t say: ‘Hey, when you get to the eighth week, you’re pain-free’.” [INT 10] “But I think the program doesn’t apply to me... You know, it’s like, geez, I thought I would discover something amazing. It’s been four years now, I’ve seen three internists, lots of doctors, and I went to a maxillofacial specialist back in September, and we’re trying to figure it out, but nobody knows what it is. I’m still waiting to find out.” [INT 2]	All interviewees mentioned they would recommend the program to someone in a similar situation, reflecting satisfaction. Participants with high expectations for very specific problems might have been less satisfied.
Total mean score of the Acceptability eScale^b^: 25.2/30 (SD 3.0)	No change required “Well, it went well. I liked it a lot, I really liked the way it was put together, the way it was presented, gradually if you like. The program is super well done, I’m going to revise it, but I wouldn’t change a thing if you asked me if there was anything to change.” [INT 1] Use of multiple media “I loved it because there were testimonials. It wasn’t just reading. I put on my headphones, and I didn’t have to read. I listened. I like to listen, and then as I filled out my sheet, I’d make notes on the paper as I listened. Sometimes I’d take breaks, put it on pause and come back.” [INT 9] Promoting access for everyone “God, yes, it’s acceptable, and everyone should have access to it.” [INT 7]	Overall, the program was well developed for our target users and proposed an appreciated gradual approach to the application of different strategies.
Of the 15 interviewees, 10 (67%) were still accessing the program or using the personal plan on an occasional to regular basis for >5 months after they finished it for the first time.	Web-based content accessibility “It helps a lot that it’s online, so you can do it when it suits you. Because for me, if I’d had to travel, you know, appointments and things like that, I’d have had a lot more trouble because it’s hard for me to go on the road, whereas here, it was much more accessible, which is really great.” [INT 12] Possible punctual use once the program is done “Then again, it’s not a program that once it’s done, it’s done, and you can’t go back to it; you can go back and look. So you can continue to use it.” [INT 10]	While the web-based format did not suit everyone, participants appreciated using it whenever they wanted and having the possibility to go back to previous lessons for a refresher. Not traveling to learn self-management strategies was a significant advantage. Had the program been offered through weekly classes at a specific time and place, some participants would have been unable to drive and attend.

^a^INT: interviewee.

^b^The Acceptability eScale has 6 items with a total score ranging from 6 to 30. Higher scores represent a high level of acceptability.

### Engagement

Of the 63 participants who consented to the study, 46 (73%) started the program, 29 (46%) finished at least 6 weeks’ lessons, and 26 (41%) completed all the lessons (Table 5).

Participants generally followed the lessons in order. Among the 19 noncompleters who did not withdraw from the study, 10 (53%) wrote back to us after receiving the email reminder. These participants provided personal reasons for not following the program according to schedule, including sickness or mental health issues among close relatives, the death of a parent, estate management, and increased symptoms. Including participants who withdrew from the study, 21 (33%) out of 63 participants were not connecting weekly or stopped at some point for reasons external to the study or the intervention. Reasons related to the program included having already applied the proposed strategies and having trouble connecting. Of the 63 participants, 5 (8%) needed help logging in independently (n=3, 60% only required information by email and n=2, 40% received phone support and succeeded with a step-by-step explanation but did not connect afterward).

**Table 5:** Participants’ weekly lessons’ level of completion (N=63).

Weekly lessons	Completed the lesson, n (%)	Partially completed the lesson, n (%)	Never initiated the lesson, n (%)
Lesson 1	42 (67)	4 (6)	17 (27)
Lesson 2	37 (59)	6 (10)	20 (32)
Lesson 3	33 (52)	6 (10)	24 (38)
Lesson 4	32 (51)	3 (5)	28 (44)
Lesson 5	30 (48)	5 (8)	28 (44)
Lesson 6	29 (46)	3 (5)	31 (49)
Lesson 7	28 (44)	4 (6)	31 (49)
Lesson 8	26 (41)	4 (6)	33 (52)

Interviewees mentioned other factors potentially undermining the engagement in completing the program. Interviewees highlighted that timing and pain acceptance played an essential role in their perseverance or lack thereof. For example, understanding that no specific cause for her pain issue might ever be found was a turning point in a participant’s proactivity:

When I was still at the stage where I thought we would find a cause, it is something I really wouldn’t have been ready to do, the program... Doing the program, really, it sort of happened right at the time I got to the stage of realizing: ‘Okay, now I’m going to stay that way. What am I going to do with this?’ I felt a lot of psychological distress. Then, looking more into this (psychological) aspect through the program, it was as if I needed to do this. I’m not done, but it’s come a long way. And also, time matters; getting used to accepting.

INT 11

For some interviewees, occasional or regular coaching would have been necessary to sustain motivation, answer questions, and revise their personal plan. Of the 63 participants, 2 (3%) mentioned their need for support and feedback:

There were ups and downs. There was no one to answer my questions. I found that very, very hard, especially the first few weeks.

INT 5

I really need someone who’ll say, “Go, we’ll do this, we’ll do that.” In writing, I’ll read, and I’ll say to myself, “Oh my god, that’s wonderful,” but I’ll do it 2 or 3 times, and then I’ll give up... But I’ve always been like that; I’ve always needed someone to push me, not in everything, but especially since I’ve been ill.

INT 9

There are participants who related less to parts of the program because their activity level was not adequately represented:

We always assume that people aren’t physically active when in pain. Or if they are, clearly, it can’t be too much. But you know, on my part, I will get injured before I stop [laughs].

INT 11

Furthermore, interviewees described factors mostly contributing to their engagement in completing the program. They expressed a connection to the program’s content and felt more hopeful, supported, and less alone listening to the multiple integrated testimonials of individuals with lived experiences applying the strategies:

For me, it was seeing lots of testimonials from many people ... that’s what really attracted me. To understand it better and to see that that’s how it is: everyone’s gone down exactly the same path I went through, and we all arrive at the same point, not being able to get out, not seeing anyone. It seemed like it was just me who was going through this. So, it gave me a boost. It also gave me a bit of confidence... I had the impression of being accompanied.

INT 14

Moreover, a participant living far from a major urban area highlighted the appeal of these short videos:

Here, in my neighborhood, there’s no program like that. I know there are places, there are meetings for people with chronic pain to chat, have a coffee, and things like that. We don’t have that here. I found it fun to listen to them and know we’re not alone in this.

INT 9

On the one hand, interviewees thought the weekly connection and review of personal objectives supported their motivation and helped them stay focused:

I guess I needed to be held by the hand for a while, and to be guided through it... There was some kind of follow-up. So you weren’t left to your own devices as much.

INT 12

On the other hand, it might have been too much for some individuals:

There were too many things. Every week there was something to do, you know. You didn’t have time to swallow the information and were already moving on to other things... Maybe it would have taken a week or two between each chapter. What made it easier was to stop for a few weeks, then think about all the information.

INT 5

There is a fine line between what feels like a comprehensive program and what feels like an overwhelming task for unsupported participants. While most interviewees showed interest in all the presented topics and perceived the lessons’ sequence as logical, gradual, and positive, specific strategies within these lessons might have lacked appeal to some participants. A suggestion was made that participants could start with their most appealing lessons after covering the pain education section. While we invited everyone to complete the tasks in order, the content was freely available and one could decide to skip parts of the program if desired.

Through the interviewee discourse, we could distinguish microlevel engagement, including the number of lessons they have followed, from macrolevel engagement, referring to the depth of involvement with the behavior change process, such as applying strategies consistently in a real-world setting [55,56]. Behavior change could be challenging, with participants facing expectations from others and their own. However, the program provided them with a better understanding of why it is beneficial to do so, and some participants made it a priority. Getting into the habit of doing something sometimes required broader or prior changes such as setting boundaries with the extended family. The notion of beginning with the easiest tasks was mentioned to underscore the initial challenge of implementing a strategy. Interviewees described the integration of certain habits into their routine as gradual, sometimes evolving over several months, without the participants realizing it. Over 5 months after finishing the program, interviewees described what they continued to apply and how some strategies became part of their routine:

Over the weeks, I really selected what had a positive impact on me. Then I do it regularly, almost every day. I’m into meditation, cardiac coherence, managing energy, stretching, and the gratitude journal. The other things, I can’t think of anything else I could do more.

INT 6

I still do so to this day, which is unusual for me. So, I thought it was really, really good... I’m more active now too, I do my exercises almost daily. I used to say to myself, “It’s no use, I won’t be able to do it. I don’t have the energy. I’m in pain. I’m out of shape.” Now I say, “Look, do it, even if it’s just two minutes today, it’ll at least be two minutes, you’ll have done it.”[INT 12]

### Preliminary Effects Outcomes

We observed an indication of improvements over time in self-efficacy, pain interference, depression, and pain catastrophizing between baseline and postintervention (Table 6). The results of the linear model corrected for the individual (treatment effects) are presented in Table 7. In addition, 24 (56%) out of 43 participants reported some level of improvement on the Patient Global Impression of Change Scale after the intervention.

From a qualitative perspective, 14 (93%) out of 15 interviewees reported a different set of effects 5 to 7 months later. These effects included, among others, shorter and less frequent pain attacks, better management of pain, higher sense of control, less comparison with life before pain, improved psychological state, more patience, less frustration and irritability, forgiveness toward oneself, higher activity level, more self-care, and increased social life.

In Table 7, we have presented the integrated results of the quantitative and qualitative phases, with the overarching theme that the various effects observed were potentially influenced by macrolevel engagements.

The effects of the lifelong task of self-management might become noticeable over time. A participant mentioned recognizing these effects several months after completing the program:

While I was going through (the program), well, you know, it was okay. In my case, it’s really like it slowly permeated me, but in a positive way, I mean.

INT 11

Therefore, conducting the interviews 5 to 7 months after program completion was deemed appropriate.

**Table 6:** Mean scores for outcome measures at baseline, postintervention, and 3-month follow-up.

Measure	Subscale or construct	Baseline (N=63), mean (SD)	Postintervention (n=43), mean (SD)	3-month follow-up (n=34), mean (SD)
Pain Self-Efficacy Questionnaire^a^	Self-efficacy	26.9 (13.8)	32.4 (13.3)	32.5 (12.4)
Brief Pain Inventory	Pain intensity	5.5 (1.6)	4.8 (1.5)	5.0 (1.6)
Brief Pain Inventory	Interference	5.7 (2.0)	4.4 (2.0)	4.7 (1.9)
Hospital Anxiety and Depression Scale	Anxiety	9.0 (4.4)	8.4 (4.2)	8.2 (4.0)
Hospital Anxiety and Depression Scale	Depression	8.5 (4.0)	7.2 (3.9)	7.5 (3.8)
Pain Catastrophizing Scale	Catastrophizing	25.3 (10.9)	20.1 (12.2)	20.5 (13.2)

^a^A higher score in the Pain Self-Efficacy Questionnaire indicates a high level of self-efficacy.

**Table 7:** Joint display of participants’ perceptions of the Agir pour moi (APM) program’s effects.

Quantitative results	Qualitative findings	Integrated analysis
Pain Self-Efficacy Questionnaire: the scores increased by 3.06 points on average after intervention (95% CI 1.26 to 4.85), *P*=.002. No effects were detected when examining postintervention and follow-up metrics.	Better self-efficacy “I’m less inclined to compare myself with my previous life. I see more of what I’m capable of doing today with the abilities I have. That’s the main thing, I think.” [INT^a^ 12] “Even though I’ve seen specialists who gave me medication, in the end, I think I got better by doing this on my own and saying to myself, Okay, I’m basically taking charge... I feel like I can control my pain peaks a bit more, and I know why I will have them.” [INT 4] “Let’s just say I’ve relearned how to gain confidence in myself and then say you’re capable, go ahead, go take a walk, you can do it.” [INT 8]	Participants’ belief in their capacity to do certain things to achieve their goal increased.
BPI^b^ pain intensity subscale scores decreased by 0.32 points on average after intervention (95% CI –0.55 to –0.10), *P*=.007. No effects were detected when examining postintervention and follow-up metrics.	No trend in pain intensity changes “I can say that my pain is less present and that it’s not all I think about anymore. So yes, for my pain, it helped a lot.” [INT 5] “It’s just that instead of being in pain for nine hours at a time, well, not only have I hardly had any attacks for six, seven months, but when it happens, well the two times it happened, it lasted two, three hours or so, then it’s stopped really suddenly, instead of lasting a whole night. So I tell you, it’s not so bad.” [INT 14] “But the pain remains the same. Sometimes when I’m doing your stuff, I’ve managed to get away for half an hour or an hour, but it comes right back.” [INT 3] “I’ve done things, like mediation and all that, but it doesn’t work.” [INT 2]	There were improvements for some interviewees and no improvements for others. While not measured with the BPI subscale, the frequency of pain crises decreased in some participants. However, as mentioned straightforwardly at the beginning of the APM, pain reduction is not the main objective of a self-management program.
BPI pain interference subscale: scores decreased by 0.78 points on average after intervention (95% CI –1.19 to –0.37), *P*=.001. No effects were detected when examining postintervention and follow-up metrics.	Less interference “I realize that the energy is coming back a little. It’s coming back. It’s not a complete crash like it used to be for me, and then I’d take a week to recover because I’d gone over the edge. Now, I don’t do that anymore ... And in the end, I do a lot more than I used to do that way.” [INT 12] “You know, I was more or less able to do some movements, some I couldn’t do at all anymore. There are some that I’ve gradually managed to recover a little, it’s not to the maximum here, but ... like putting on my shoes.” [INT 15] “Well, I fall asleep faster when I do these exercises. Because I always try to go to bed at the same time, and sometimes sleep doesn’t come. So I tell myself I’m going to bed anyway and do some breathing exercises. Then I fall asleep, which doesn’t take long.” [INT 15]	Reducing pain interference translates into a gain in energy, a more stable ability to perform tasks, a new capacity to do movements, increased social activities, and better sleep.
HADS^c^ depression subscale: scores decreased by 0.73 points after intervention (95% CI –1.21 to –0.25), *P*=.005. No effects were detected when examining postintervention and follow-up metrics.	Positive change in depressive state “It had been seven years since I’d stopped putting any effort into it and let myself fall, so it...no, no, it whipped me, and then I seemed to become a bit like myself again. I was letting myself go, then it was like: okay, go, I’ve been sinking for seven years, and now it’s time to get back on. It gave me a good boost. ... My mood has changed. I seem to be less, sorry about my French, but I’m less (swear) angry all day long.” [INT 14] Better mood “how can I put it, patience, my patience came back, better than when I couldn’t do my things. Yes, yes, that, I’ve made some gains.” [INT 15] “It’s also psychological, you know, like being less on edge in my head, I was a lot like (swears), I can’t do this anymore, I can’t. So I’m in a better mood with the kids. ... It also has a lot to do with irritability, because you know when you’re in pain, you’re always irritable, so if I’m in less pain, I’m less irritable, and I’m more likely to want to go outside with them.” [INT 11]	Most interviewees did not explicitly talk about depression but many reported being in a better mood, being less frustrated, and being less irritable.
HADS anxiety subscale: nonsignificant score decrease of 0.37 points after intervention (95% CI –0.85 to 0.12), *P*=.14. No effects were detected when examining postintervention and follow-up metrics.	Anxiety “it’s really taken my anxiety level about it down a notch.” [INT 11]	Following the program does not appear to impact the anxiety state. Only 1 interviewee briefly mentioned a decrease.
Pain Catastrophizing Scale: scores decreased by 2.83 points after intervention (95% CI –4.35 to –1.30), *P*=.001. No effects were detected when examining postintervention and follow-up metrics.	Less panic “For me, what was a real game changer ... was also realizing that most people in this condition tend to have the same thoughts; thinking, for example, that there’s something perhaps serious hidden behind it, thinking that it will never stop, that you could die from it, and all that. It was good for me, because these are patterns I really have. Then, I thought of myself more like a normal person. ... The panic I used to feel about my pain has almost completely disappeared, and I realize how much I can change my situation myself.” [INT 11]	The testimonials and theoretical content helped normalize some participants’ catastrophic thoughts and guide them in confronting those and adopting more adapted views. Following the program can reduce catastrophizing.

^a^INT: interviewee.

^b^BPI: Brief Pain Inventory.

^c^HADS: Hospital Anxiety and Depression Scale.

## Discussion

### Principal Findings

This paper described a pilot, mixed methods study assessing feasibility and acceptability and exploring the preliminary outcomes of a new self-directed, web-based program for chronic pain among adults awaiting superspecialized services. Collaborating with patient partners experiencing diverse types of pain was a key strategy enabling us to align the program closely with the varied needs and expectations of our wide-ranging audience. We opted for a simple yet attractive layout, providing clear instructions and features to aid those with attention and concentration challenges.

### Feasibility, Acceptability, and Engagement

Chronic noncancer pain affects individuals in different ways and to different degrees. Those awaiting tertiary services in Canada experience severe impairments and present with a poor biopsychosocial profile [2]. To specifically recruit these individuals who are not yet patients at the center of expertise in chronic pain management, we could not directly reach out to them. The hospital’s archives had to send them invitation letters. Estimating the response rate in such a specialized context proved challenging because we lacked a benchmark for our expectations. However, a study by Thiblin et al [57], which involved a comparable internet-administered, CBT-based self-help intervention, achieved an 11% enrollment rate by sending out 509 invitation letters. Considering the documented high dropout rates in similar trials [19,58], we anticipated that 500 invitations would suffice to ensure a minimum of 30 participants responding to questionnaires at all 3 time points. Our enrollment rate for a 3-month recruitment period was similar to or better than those of studies recruiting in tertiary pain treatment facilities [59,60]. The consent rate among potentially eligible adults was acceptable. However, because 4 (6%) out of 63 participants exited the study for surgery purposes and 3 (5%) others for significant changes in their medication, we might need to reconsider how we address these eligibility criteria during the phone interview. Furthermore, considering there are between 1000 and 1500 individuals awaiting services from the center of expertise and that they all would not meet our eligibility criteria, our response rate, although similar to those observed in other studies, would require conducting a future full trial across multiple centers.

Our study yielded comparable data collection results to other web-based intervention pilot trials with approximately 50% (35/63) of response at 3-month follow-up [61,62]. While studies with higher financial incentives (US $25-$80 per assessment) during visit assessments or initial motivational interviews had better response rates [60,63-66], we purposefully chose to stay as close as possible to real life where no incentives are offered. We received no negative feedback on the number or length of questionnaires. However, sending email reminders to participants who did not log in or complete the questionnaires at the appropriate time was suboptimal. Making phone calls in addition to email reminders might have provided us with reasons for disengagement and ensured participants received the reminders.

Some interviewees highlighted that the log-in process was not intuitive, leading us to consider modifying this aspect before conducting a full trial. The log-in was essential to the research project but is not part of APM itself and will not apply in real life. Once logged in, accessing APM at their most convenient time and place was a significant asset for our participants, consistent with patients’ preferences [21].

APM’s codevelopment with health care professionals and people with lived experience of chronic pain allowed a tailored approach to this population’s needs and preferences in a web-based self-management program [32]. The interviewees’ description of APM aligned with the acceptability score as a globally acceptable, easy-to-use, and engaging program. We based our 24 (80%) out of 30 threshold score for the Acceptability eScale based on the study by Tariman et al [38], suggesting that 80% of the highest possible summary score indicates good program acceptability. However, a score <24 would not automatically deem the program unacceptable. We must examine individual item scores to assess specific program weaknesses. All items scored ≥4 (≥80%) out of 5, as show in Table 4, indicating no significant flaws requiring major modifications. Minor improvements we could make include adding testimonials from highly active individuals with chronic pain who learned to pace themselves and mentioning that lessons are preferably followed in order but can also be explored based on personal preferences after completing week 1. APM effectively promoted behavioral change, guided participants in taking action, and served as a reference in the longer term.

While participants’ weekly lessons’ level of completion was lower than anticipated, it was consistent with what had been observed in other feasibility and pilot studies [62,67-69] and could be explained mainly by reasons external to the study. Our qualitative results alleviated concerns about potential flaws and did not point toward questioning the participant’s appeal to the program. Overall, we are confident that the program and trial procedures are both feasible and acceptable.

### Preliminary Effects

We explored pre- and postintervention effects as preliminary indications of potential changes in self-efficacy, pain intensity and interference, anxiety, depression, and catastrophizing. Findings yielded relevant results, but these should be interpreted cautiously.

Nevertheless, the qualitative interviews pointed in the same direction as our preliminary quantitative findings. Furthermore, these aligned with the results of a meta-analysis suggesting that following internet-delivered, CBT-centered interventions for chronic pain can lead to small significant improvements in pain interference and intensity, depression, anxiety, self-efficacy, and catastrophizing, with greater treatment effects in anxiety, pain interference, and intensity in guided compared to unguided interventions [34]. Therefore, depending on their perceived importance of change and self-efficacy, individuals with chronic pain may require additional support in reaching readiness to make sustainable changes.

Because we still did not know precisely what clinical, intervention, and study characteristics positively impacted the effects of unguided CBT-based self-management programs for chronic pain, APM offered several self-management strategies [70]. However, we did not expect participants to implement all of them once they completed the program. Participants used this program as a toolbox, as mentioned by an interviewee.

No adverse events were reported throughout the course of this study.

### Limitations

This study has some limitations. First, we cannot make definitive statements regarding APM’s effects without an appropriate control group, randomization process, and sample size. Indeed, we neither designed nor appropriately powered this feasibility trial to test a specific hypothesis [36]. Furthermore, while the participants presented various pain conditions, most of the female participants were White, as this is the case in similar trials [18,71,72], and were all attached to a single center. Recruitment strategies of a future randomized controlled trial should focus on attracting a broader representation of individuals with chronic pain in terms of gender, ethnicity, and health care institutions. In Quebec City, where our study was conducted, <10% of the population identified as members of a visible minority group in 2021. Expanding our research to cities with greater ethnic diversity could enhance our sociodemographic data and improve the relevance of our findings. Shifting to web-based recruitment methods might allow us to create tailored invitation messages for specific demographic groups, using a casual design and images, instead of overwhelming potential participants with excessive written information. In our feasibility study, we had to use standardized letters to recruit participants from the waitlist of the center of expertise in chronic pain management. We acknowledge the need to adopt more flexible parameters for future large-scale studies. Furthermore, we might adjust our eligibility criterion, not limiting participation to those awaiting specialized services. This broader criterion could yield a more diverse sample, aligning with our aim to reach a wider demographic. However, we are mindful of the potential impacts on adherence and user satisfaction this broader criterion might pose. Nevertheless, these adjustments reflect our dedication to conducting a comprehensive and inclusive trial, ultimately contributing to a more nuanced understanding of chronic pain management. This may lead us to unforeseen modifications in our program.

Despite widespread internet access in Canada, disparities in internet speed, affordability, and digital literacy persist. APM, being exclusively web-based, poses a limitation in reaching individuals from remote regions and Indigenous communities as well as those in low-income households, older adults, and individuals with disabilities. These groups are disproportionately affected by the digital divide in Canada, making it challenging for them to access our program.

### Future Direction

This study provides an initial understanding of APM’s potential benefits for this group of individuals with chronic pain awaiting specialized services. Through the interviews, we acknowledged we had not captured the effects on the temporal aspect of pain, such as shorter and less frequent pain attacks, which were crucial for some participants. Therefore, we could consider adding measures capturing these aspects in a future trial.

Developing a web- and evidence-based, patient-centered, free-of-charge, user-friendly, and French self-management program for chronic noncancer pain represents a potential response to the clearly expressed needs of individuals with this condition. Although the literature increasingly emphasizes the importance of personalization in eHealth, our limited financial resources hindered us from incorporating advanced features. We deliberately chose to focus on fundamental aspects and prioritize what we could offer and support in the long term, establishing the groundwork for a web-based program that could potentially evolve. As a result, the current version did not include personalized features, but it was still perceived as usable and useful. It will be essential to document how the program’s implementation makes it possible to respond quickly and more equitably to some of the needs of patients waiting for services or who live far from large centers. APM is currently being used without restrictions in other French-speaking regions and countries. Anyone can use it freely, but a potential hurdle faced when using it abroad pertains to adapting to the accents in testimonial videos and Quebec-specific expressions.

### Conclusions

The study ﬁndings provided preliminary evidence that the APM program and research methods were both feasible, as suggested by perceived acceptability and engagement. Furthermore, it provided preliminary indications of potential improvements in self-efficacy, pain intensity, interference, depression, and catastrophizing. The study yielded essential results to undertake a future complete trial.

## Appendix

Multimedia Appendix 1 CONSORT-eHEALTH checklist (V 1.6.1).

## Abbreviations

APM: Agir pour moi
CBT: cognitive behavioral therapy
CHU: University Hospital Centre
CONSORT-EHEALTH: Consolidated Standards of Reporting Trials of Electronic and Mobile Health Applications and Online Telehealth
REDCap: Research Electronic Data Capture

## Data Availability

The data sets generated during and analyzed during this study are available from the corresponding author on reasonable request.
